# Altering DNA Repair to Improve Radiation Therapy: Specific and Multiple Pathway Targeting

**DOI:** 10.3389/fonc.2019.01009

**Published:** 2019-10-10

**Authors:** Julian Biau, Emmanuel Chautard, Pierre Verrelle, Marie Dutreix

**Affiliations:** ^1^Institut Curie, PSL Research University, Centre de Recherche, Paris, France; ^2^UMR3347, CNRS, Orsay, France; ^3^U1021, INSERM, Orsay, France; ^4^Université Paris Sud, Orsay, France; ^5^Université Clermont Auvergne, INSERM, U1240 IMoST, Clermont Ferrand, France; ^6^Radiotherapy Department, Université Clermont Auvergne, Centre Jean Perrin, Clermont-Ferrand, France; ^7^Pathology Department, Université Clermont Auvergne, Centre Jean Perrin, Clermont-Ferrand, France; ^8^U1196, INSERM, UMR9187, CNRS, Orsay, France; ^9^Radiotherapy Department, Institut Curie Hospital, Paris, France

**Keywords:** DNA damage, repair systems, radiotherapy, radioresistance, inhibition

## Abstract

Radiation therapy (RT) is widely used in cancer care strategies. Its effectiveness relies mainly on its ability to cause lethal damage to the DNA of cancer cells. However, some cancers have shown to be particularly radioresistant partly because of efficient and redundant DNA repair capacities. Therefore, RT efficacy might be enhanced by using drugs that can disrupt cancer cells' DNA repair machinery. Here we review the recent advances in the development of novel inhibitors of DNA repair pathways in combination with RT. A large number of these compounds are the subject of preclinical/clinical studies and target key enzymes involved in one or more DNA repair pathways. A totally different strategy consists of mimicking DNA double-strand breaks via small interfering DNA (siDNA) to bait the whole DNA repair machinery, leading to its global inhibition.

## Introduction

Radiation therapy (RT), in conjunction with surgery and systemic therapies (chemotherapy, targeted therapies, immunotherapy…), is a cornerstone of cancer care. About 50% of cancer patients receive RT (1). The primary objective of RT is to increase the amount of radiation delivered to the tumor to ensure local control and reduce the amount of radiation in adjacent healthy tissues. Advanced developments such as image-guided RT (IGRT) or intensity-modulated RT (IMRT) have led to the enhancement of this therapeutic ratio (2). Despite such improvements, many patients still experience local recurrence of the disease after RT. Clinical factors such as tumor stage, frequently associated with increased hypoxia, can explain some of the failures, but it is clear that biological characteristics play a key part in successful treatment (3–5). RT-induced cell death is mostly due to DNA damage, especially to double-strand breaks (DSBs) (6, 7). Consequently, tumor cells with highly efficient DNA repair are radioresistant (8), whereas deficiencies in pathways that repair DSBs are particularly detrimental to the cells (9). Therefore, therapies that inhibit the DNA repair machinery have the potential to enhance RT efficacy (10, 11). Inhibiting DNA repair offers an opportunity to target genetic differences between tumor and normal cells, as DNA repair is often dysregulated in tumor cells (10, 12–14). Tumor cells divide rapidly because of unregulated cell cycle control. Thus, they have less time to repair DNA damage as compared to normal cells that are not dividing or will stop dividing after activation of key checkpoints induced by RT (15, 16). Beside altered cell cycle control, cancer cells may also present defects in their DNA repair system, inducing dependence on specific repair pathways and/or overexpression of alternative pathways (16, 17). Furthermore, cancer cells often develop under stress conditions, thus raising the frequency of endogenous DNA damage (18, 19). This review will firstly focus on the distinct categories of DNA lesions induced by RT and the DNA repair pathways required for their repair. Subsequently, it will present the approaches that are currently being developed to enhance RT efficacy by modulating DNA repair.

## RT-Induced DNA Damage Response

DNA lesions induced by RT activate the DNA damage response (DDR), which essentially involves post-translational modifications of proteins to activate downstream signaling pathways (20). DDR is based on an intricate network of proteins that work together to manage DNA repair and cell cycle coordination. DDR interrupts the cell cycle, thereby inhibiting the spread of DNA damage to daughter cells and facilitating repair. Cell division arrest is mainly controlled by the checkpoint kinases CHK1 and CHK2, which are activated by the phosphatidylinsositol-3-kinases (PI3K) of the DDR machinery (15). DDR signaling is also essential for triggering apoptosis when repair is unsuccessful, notably through modifications to the p53 protein (20).

RT induces a variety of DNA lesions. Approximately 10,000 damaged bases, 1,000 single strand breaks (SSBs) and 40 DSBs are produced per gray per cell (21, 22). Such lesions, if not corrected, can lead to cell death by mitotic catastrophe and apoptosis. DSBs are the most lethal to the cells despite their low proportion, as one single unrepaired DSB can trigger cell death (7). DSBs are produced directly and indirectly by RT. Indirect DSBs most often occur during replication if the initial damage is unrepaired. As an example, when a replication fork encounters an unrepaired SSB, the fork is blocked and leads to the conversion of this SSB into a DSB (10, 23, 24). The resulting DSB can directly trigger cell death or activate DDR, which induces cell cycle arrest and promotes DNA repair. This repair is usually error-free, allowing the cell to survive without genetic consequences. It can also be error-prone, leading either to cell death if the error is not viable or mutation and chromosomic aberrations (25).

## DNA Repair of RT-Induced Damage

**Figure 1:**
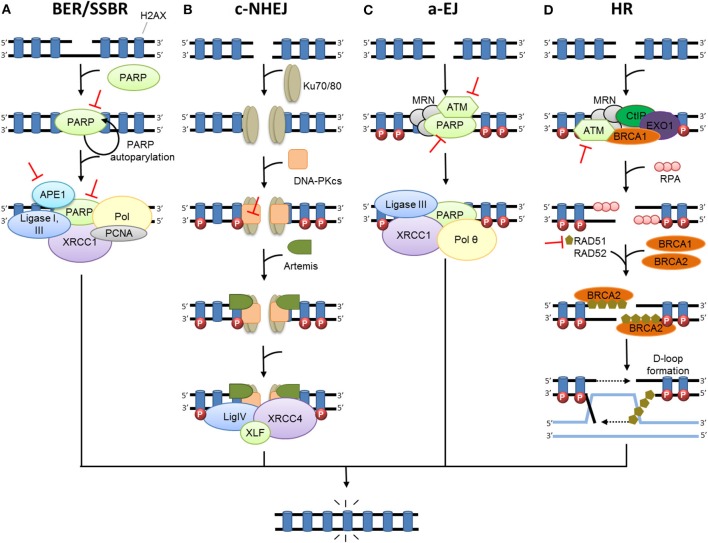
DNA damage repair after radiation therapy. In irradiated cells, a number of DNA lesions are induced including single (SSB) and double-strand breaks (DSB). **(A)** SSBs are corrected by the part of base excision repair (BER) known as single-strand break repair (SSBR). The binding of PARP to SSB activates its auto-PARylation and leads to the recruitment of BER/SSBR proteins including AP endonucleases (APE1), XRCC1 (helper protein), PCNA, FEN, PNK, and DNA polymerases (Pol; damage processing) and DNA ligases. **(B)** In c-NHEJ, DSB is recognized by the Ku80-Ku70 heterodimer, which leads to DNA-dependent protein kinase catalytic subunit DNA-PKcs recruitment, gathering the DNA-PK complex and activating its kinase activity. This leads to the involvement of repair proteins (XRCC4, DNA ligase IV and others), which perform the processing and final junction reaction. **(C)** When c-NHEJ is impaired, an alternative pathway called a-EJ (alternative EJ) takes place and involves mainly PARP1, XRCC1, ligase III, MRN complex and the DNA polymerase θ. **(D)** In HR, after ATM activation, the DSB site is bounded by the MRE11-RAD50-NBS1 complex (MRN). The consequence is the phosphorylation of a set of targets including H2AX (γ-H2AX), localized at the site of DSB. HR uses the sister chromatid as a model to repair DSB. First, the resection of the DNA at DSB results in a 3′ single-strand DNA, which is then coated by proteins of replication A (RPA). Subsequently, proteins of the RAD family are recruited and mediate the invasion of the homologous strand of the sister chromatid, leading to the formation of Holliday junctions. DNA polymerases can then synthetize across the missing regions. The Holliday junctions are finally resolved by cleavage and followed by ligation of adjacent ends. 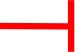 Represents inhibitors of DNA repair in preclinical or clinical development.

Following RT, damaged bases induced by oxidative stress are repaired by the base excision repair pathway (BER) (26–31). In BER, damaged bases are excised by DNA glycosylases, resulting in apurinic (AP) sites. Subsequently; these AP sites are cleaved by apurinic endonuclease 1 (APE1) or an AP-lyase activity, leading to SSBs. SSBs are repaired by the part of the BER pathway called single strand break repair (SSBR) (Figure 1A) (12, 32). Either short-patch or long-patch SSBR can then proceed, depending on several factors such as type of lesion and cell cycle state. Single nucleotide insertion by DNA polymerase (Pol) β and ligation by DNA ligase III are described as short-patch SSBR, and interact with the protein X-ray repair cross-complementing 1 (XRCC1). Long-patch SSBR involves the removal of a larger DNA segment, which requires several DNA replication factors such as proliferating cell nuclear antigen (PCNA), Pol δ/ϵ, flap endonuclease 1 (FEN1), and DNA ligase I. Concerning SSBs detection, poly-ADP-ribose polymerase (PARP1 or PARP2) are required (28, 33–37). The binding of PARP to SSB activates its auto-PARylation, and leads to the recruitment of BER/SSBR proteins. PARP-1 was also reported as a regulator of DNA repair gene expression through the E2F1 pathway (38). Unrepaired SSB or a damaged base can block the replication forks, resulting in fork collapse and DSB (23). The great majority of oxidative damage induced by ionizing radiation is corrected by BER. However, under hypoxic conditions, IR causes the formation of cyclodeoxynucleosides that can be only removed by nucleotide excision repair (NER). Several results suggest that NER may be involved in the repair of oxidized DNA damage. In addition, ionizing-radiation breast cancer risk has been related to polymorphism in ERCC2 (one of the main NER enzymes) (39).

Two major pathways repair DSBs: homologous recombination (HR) and non-homologous end joining (NHEJ) (40). However, both mismatch repair (MMR) and NER pathways have been reported to affect both HR- and NHEJ-mediated DSB repair efficacy to a lesser extent (41). The formation of DSBs triggers the activation of three key enzymes from the PIKK family: ataxia telangiectasia mutated kinase (ATM), ATM-related kinase (ATR), and DNA-dependent protein kinase (DNA-PK). This leads to the phosphorylation of many proteins, signaling damage and initiating DNA repair. One of the early steps is the phosphorylation of histone H2AX (γ-H2AX), which signals the presence of DSB to repair proteins where they aggregate in ionizing radiation-induced foci (IRIF) (42). Besides their crucial roles in DDR signaling, the kinases ATR and ATM are also involved in maintaining replication fork stability (14) and fork reversal in case of fork-stalling lesions, notably through SMARCAL1 (43).

In mammalian cells, c-NHEJ (classical NHEJ, Figure 1B) is the most efficient DSB repair mechanism. It acts by directly ligating the broken DNA ends (44). c-NHEJ can occur during the entire cell cycle. It is frequently accompanied by small deletions at the repair break site and is considered to be the main cause of DSB error-prone repair. The first step of NHEJ is the binding of the heterodimer Ku70/Ku80 at the end of the DSB (45), allowing the recruitment of catalytic subunit DNA-PKcs forming the protein complex DNA-PK (46). DNA-PK, bounded to DNA, is activated and phosphorylates numerous proteins including H2AX (47), Artemis (48), X-ray repair cross- complementing 4 (XRCC4) and ligase IV complex (49), and XLF (XRCC4-like factor) (50) that are recruited on the site of the DSB and participate in its repair. When c-NHEJ is impaired, an alternative pathway called a-EJ (alternative EJ) or MHEJ (Micro Homology End Joining) (Figure 1C) is activated (51). At the initial breaking site, a deletion of 5–25 nucleotides is necessary to reveal micro-homologies to realize a-EJ (52), while a maximum of 4 deleted nucleotides is necessary for c-NHEJ (44). The micro-homologies that are slightly longer in the case of a-EJ could partly explain the higher number of large deletions and other genomic rearrangements that occur (53, 54). The a-EJ pathway is differentiated from c-NHEJ by the fact that it is independent of Ku proteins (52). a-EJ involves mainly PARP1, XRCC1, ligase III (LIGIII), and the MRE11/RAD50/NBS1 (MRN) complex (55, 56). DNA polymerase theta (Pol θ or PolQ) is specifically involved in nucleotide incorporation in the a-EJ mechanism through the TMEJ (theta-mediated end joining) pathway (57).

HR is an alternative pathway for repairing DSBs that uses the sister chromatid as a model, restricting this mechanism to the S and G2 cell cycle phases (Figure 1D) (40). HR is the most conservative and least error-prone repair mechanism. It necessitates the presence of BRCA proteins, defects of which increase susceptibility to breast or ovarian cancer. The DSB site is bounded by several factors such as the MRN complex, EXO1 (exonuclease 1), DNA2-BLM (Bloom syndrome), BRCA1 and CTIP (CtBP-interacting protein) that contribute to DNA resection and formation of a 3′ single-strand DNA (58–60), which is then coated by proteins of replication A (RPA). After the RPA protein's displacement by RAD51, BRCA2 together with the localizer of BRCA2 (PALB2), RAD54, and BARD1 (BRCA1-associated RING domain protein 1) mediates the nucleoprotein filament invasion of the homologous strand of the sister chromatid and creates the “D-loop” (61). DNA polymerases can then synthetize across the missing regions. The resulting Holliday junctions are finally resolved by cleavage and followed by ligation of adjacent ends (62). However, HR can sometimes be error-prone, especially if template switching occurs, e.g., in repeat sequences (63).

The choice between the two major mechanisms for DSB repair (NHEJ and HR pathways) seems to be linked to several factors such as cell-cycle phase, chromatin context, or availability of key actors such as the Ku complex, 53BP1 or RAD51 (64, 65).

## Current Strategies Involved in DDR Inhibition in Combination With RT

### Targeting Key Enzymes Involved in a Specific DNA Repair Pathway

#### Inhibiting BER/SSBR

BER and SSBR pathways repair damaged bases and SSBs. Inhibiting BER/SSBR may lead to unrepaired damages that are converted to DSBs when encountering a replication fork (23). Therefore, in cells already defective for HR, such as BRCA^−/−^ breast or ovarian cancer tumors, the inhibition of BER by PARP inhibitors leads to unrepaired DSBs and cell death (14). This effect, called synthetic lethality, has been extensively described (17, 66, 67) and studied in numerous clinical trials (68–70). Since the majority of RT-induced damages are repaired by BER/SSBR, inhibition of this pathway should highly sensitize cells to RT even in HR-proficient cells (29). The preclinical evaluation of PARP inhibitors has shown enhanced RT efficacy both *in vitro* and *in vivo* (71–73). Several PARP inhibitors have already been tested in or entered into numerous clinical trials in association with RT for brain metastases, ovarian cancer, breast cancer, rectal cancer, or glioblastoma, among others (Table 1). However, early data did not demonstrate convincing and coherent proof of synergy, although neither did they demonstrate unexpected toxic effects (74, 75). Another strategy for the inhibition of BER/SSBR is the development of APE1 inhibitors. APE1 is crucial for BER/SSBR and is commonly overexpressed in cancer cells (80, 81), giving to this strategy some tumor specificity. APE1 inhibitors have shown efficacy in combination with RT in preclinical studies (82). Lucanthone, an APE1 inhibitor, combined with temozolomide, has recently been tested in a phase 2 clinical trial in glioblastoma (Table 1). The results are not yet published.

**Table 1:** Examples of clinical trials of inhibitors of the DNA damage response in combination with radiation therapy.

**DNA repair pathway(s)**	**Target**	**Inhibitor**	**Cancer type**	**Phase**	**References/trial identifier**
**Targeting key enzymes involved in a specific pathway**					
BER/SSBR	APE1	Lucantone	Glioblastoma	Phase 2	NCT01587144
	PARP	Iniparib	Glioblastoma	Phase 1/2	NCT00687765
			Brain metastases	Phase 1	NCT01551680
		Veliparib	Rectal	Phase 1	NCT01589419
			Breast	Phase 1	NCT01477489
			Peritoneal carcinomatosis	Phase 1	(74)
			Brain metastasis	Phase 2	(75)
NHEJ	DNA-PK	M3814	Solid tumors	Phase 1	NCT02516813
		M3814	Rectal	Phase 1b	NCT03770689
		M3814	Solid tumors	Phase 1	NCT03724890
HR	RAD51	Imatinib	High grade glioma	Phase 1/2	(76)
**Targeting key enzymes involved in multiple pathways**					
NHEJ/HR	ATM	AZD1390	Brain tumors	Phase 1	NCT03423628
	ATR	AZD6737	Solid tumors	Phase 1	NCT02223923
		M6620	Esophageal	Phase 1	NCT03641547
		M6620	Head and neck	Phase 1	NCT02567422
		M6620	Brain metastases	Phase 1	NCT02589522
**Targeting chromatin dynamics via epigenetic modifications**					
	HDAC	Vorinostat	Gastrointestinal	Phase 1	(77)
			High grade glioma	Phase 2/3	NCT01236560
		Valproic acid	Cervical	Phase 2	(78)
**Baiting DNA-break recognition**					
BER/SSBR NHEJ/HR	PARP DNA-PK	Dbait	Melanoma	Phase 1	(79)
**Targeting cell cycle checkpoints**					
	CHK1/2	Prexsertib	Head and neck	Phase 1	NCT02555644
	WEE1	Adavosertib	Glioblastoma	Phase 1	NCT01849146
		Adavosertib	Cervical	Phase 1	NCT03345784
		Adavosertib	Head and neck	Phase 1	NCT03028766

#### Inhibiting NHEJ

DNA-PK, a key enzyme in NHEJ, is a member of the PI3K family that performs a central role in many cellular functions (83). Selective DNA-PK inhibitors have led to radiosensitization in preclinical studies (84–86). Three phase 1 trials are currently testing the safety and tolerability of a DNA-PK inhibitor (M3814) in combination with palliative RT +/- immunotherapy in advanced solid tumors (NCT02516813 and NCT03724890) and curative-intent radiotherapy in locally advanced rectal cancer (NCT03770689) (Table 1). Such strategies, which are not based on a selective effect on the tumor, are considered promising by some (14) though they have been criticized by others (87). Early reports of the clinical combination of M3814 and palliative RT showed enhanced normal tissue reactions including dysphagia, prolonged mucosal inflammation/stomatitis, and skin injury (87, 88). Inhibition of Ku subunits could also result in reduced DNA-PK activity and NHEJ. This is consistent with the existing data reporting that shRNA depletion of Ku70 or Ku80 showed cytotoxicity and radiosensitization in pancreatic cancer cells (89, 90). CC-115, a dual inhibitor of DNA-PK and mammalian target of rapamycin (mTOR), is being tested; preliminary anti-tumor activity has been reported, although whether these responses are attributable to activity against DNA-PK or mTOR is unclear (14, 91). A phase 1 trial testing CC-115 in combination with RT and temozolomide in the treatment of glioblastoma is ongoing (NCT02977780). NHEJ can also be indirectly inhibited via the EGFR pathway (see below).

#### Inhibiting HR

Cancer cells are known to be more proliferative than normal cells (92). Inhibitors of replication-associated processes such as HR exploit this specificity to enhance the therapeutic ratio. Nevertheless, there are few specific inhibitors of HR. It has been shown that RAD51 expression and functional HR can be reduced using imatinib during experimental RT, leading to increased radiosensitization (13, 93). Indirect inhibition of HR can also be obtained via cell cycle checkpoint targeting (see below).

### Targeting Key Enzymes Involved in Multiple Repair Pathways

DNA damage detection and signaling is the first step common to all DNA repair pathways. Acting on this step will alter several pathways. Therefore, several approaches have been tested to disable part or all of the DNA damage recognition/signaling steps.

#### Inhibiting ATM

ATM is one of the key enzymes in DNA damage signaling of DSBs for HR but also NHEJ (94). Defective cells in ATM are extremely radiosensitive, independent of their p53 status (95). ATM inhibitors have shown radiosensitization in preclinical studies (96–98). A single 15Gy RT dose suppressed tumor growth in a preclinical model when ATM was deleted in cancer cells vs. when deleted in endothelial cells (99), underlining the interest in testing ATM inhibitors in combination with highly conformal RT. Like DNA-PK, ATM is part of the PI3K family and has many cellular functions. A phase 1 study is currently testing an ATM inhibitor (AZD1390) in combination with RT in brain tumors including glioblastoma and brain metastases (NCT03423628). Indirectly, inhibition of the TGFβ-signaling or mitogen-activated protein kinase (MAPK) pathway can lead to reduced ATM activation and increased tumor cell radiosensitivity through reduced DSB repair (100–102).

#### Inhibiting ATR

ATR is a critical kinase that is activated in reaction to replication stress and blocked replication forks. ATR is one of the key enzymes in DNA damage signaling of DSBs (103). Cancer cells, which exhibit relatively elevated levels of replication stress, are more susceptible to dependence on ATR signaling for survival (104). An ATR inhibitor (AZD6738) has given encouraging preclinical results (67, 105) and is currently in phase I trials as monotherapy or in combination with olaparib, RT (NCT02223923), carboplatin and immunotherapy agents. Another ATR inhibitor (M6620) is being tested in three phase 1 trials with radiotherapy in esophageal cancer (NCT03641547), locally advanced head and neck squamous cell carcinoma (NCT02567422) and brain metastases (NCT02589522).

#### Inhibiting MRN Complex

Mirin is an inhibitor of MRE11 endonuclease and thus of HR function. However, MRE11 is also upstream of NHEJ, and so mirin inhibits NHEJ and its effects are not specific to HR (16, 106, 107). It might be of particular interest in combination with RT.

#### Baiting DNA Breaks Signaling

This approach developed recently is represented by the molecules called Dbait/AsiDNA™. Dbait/AsiDNA™ consist of double-strand oligonucleotides that mimic DNA strand breaks and consequently bind and trap the signaling and repair proteins DNA-PK (24, 108, 109) and PARP (110), leading to inhibition of both SSB and DSB repair. In preclinical studies, the proof of concept that a RT-Dbait association could be used in treating melanoma (24) and high-grade glioma (111) has been established. A first-in-man phase 1 trial was conducted combining Dbait/AsiDNA™ in combination with palliative RT in in-transit metastases of melanoma (79) (Table 1). In this trial, no dose-limiting toxicity was reported and the maximum tolerated dose was not met.

### Targeting Chromatin Dynamics via Epigenetic Modifications

Epigenetics is an emerging field in cancer biology (112). It focuses on functionally relevant genome modifications that do not modify the nucleotide sequence. Such modifications include DNA methylation or histone modifications that may regulate gene expression but do not alter the associated DNA sequence. These modifications could also affect DNA repair ability. The loss of ARID1A, a piece of the SWI/SNF chromatin remodeling complex, was recently reported to induce a selective vulnerability to combined RT and PARP inhibitor therapy (113).

#### Inhibiting Histone Deacetylases (HDACs)

HDAC inhibitors are epigenetic therapeutics. They have the capacity to lower RT-induced damage repair both at the level of damage signaling, via inhibition of the ATM or MRN complex, and by directly affecting proteins involved in NHEJ and HR (112, 114–117). Several clinical trials have been carried out for various cancer types (77, 78) (Table 1).

### Inhibition of Kinases Involved in DDR-Related Survival Pathways

Sorafenib, a multi-kinase inhibitor, is currently utilized in the clinic for the treatment of hepatocellular and renal cancers. It inhibits MAPK signaling together with additional intracellular Ser/Thr kinases, leading to both NHEJ and HR inhibition. Sorafenib has shown a radiosensitization effect in preclinical studies (118). Dasatinib is another multi-kinase inhibitor inhibiting ABL and SRC tyrosine kinases. In preclinical studies, it has shown a radiosensitization effect partly due to blocking of DNA repair pathways involved in DSB repair (119). Sorafenib and dasatinib are clinically evaluated in association with RT. Because of their large spectrum of targets, most of these inhibitors may show high toxicity, which prevents them from being used at the dosage required to be efficient in combination with RT in the management of aggressive tumors that overexpress some of their targets (120, 121).

After RT, EGFR has been found to translocate into the nucleus and modulate DNA repair (especially NHEJ) through association with DNA-PKcs (122, 123). A current in-clinic approach is using the monoclonal antibody cetuximab to inhibit this nuclear translocation of EGFR. Cetuximab combined with RT has improved patients' overall survival in a phase III trial in head and neck cancer (124). Furthermore, EGFR signaling may be interrupted by small-molecule tyrosine kinase inhibitors such as erlotinib or gefitinib, especially in the case of specific EGFR mutation; these are currently being tested in combination with RT (125, 126).

### Targeting Cell Cycle Checkpoints

Checkpoint dysfunction represents a common molecular defect acquired during tumorigenesis (15, 127), underlying the importance of its regulation in cancer development. Interfering with cell cycle checkpoint signaling is an alternative approach to modulating DNA repair activity and potentially improving the therapeutic ratio. The induction of DNA lesions by RT in normal cells stops their progression in the cell cycle, thereby avoiding the accumulation of other lesions and their damaging effects (20). This cell cycle arrest is subtly correlated with DNA repair to fine-tune cell cycle restart with the disappearance of damage. In cancer cells with an altered G1 checkpoint, cell cycle progression goes on relentlessly and, as a result, the removal of the G2 block increases unrepaired damage and its transfer to the daughter cells. This finally causes the loss of essential genetic material and cell death, a process that strengthens checkpoint inhibition strategies. Combination of RT with a dual CHK1 and CHK2 inhibitors (AZD7762 and prexsertib) showed a radiosensitization effect with an increase in mitotic catastrophe in different cancer cell lines and xenografts (128–130). A phase 1b trial was completed that combined prexsertib with RT and cisplatin or cetuximab in locally advanced head and neck cancer (NCT02555644), the results of which are not yet published. However, in addition to checkpoint activation, CHK1 is also involved in HR (131, 132) and it is uncertain if this is only a result of checkpoint inhibition or if it is partly due to HR inhibition (133).

Another target is the WEE1 kinase, which has been shown to be a major regulator of the G2-M checkpoint (134). This tyrosine kinase inhibits the entrance in mitosis by adding an inhibitory phosphorylation to Cdc2 (the human homolog of tyrosine kinase 1[Cdk1]) to tyrosine 15 (Y15). As a consequence, the Cdc2/cyclin B complex becomes inactivated, which stops the cells in G2-M and allows DNA repair. Preclinical studies have shown the potential use of WEE 1 inhibitors as radiosentizers (135, 136). Several ongoing clinical trials are testing WEE1 inhibitors with RT. In addition, several phase 1 trials are currently testing the WEE1 inhibitor adavosertib (AZD1775) in combination with RT and temozolomide in the treatment of glioblastoma (NCT01849146), with RT and cisplatin in cervical, vaginal or uterine cancer (NCT03345784), or in combination with RT and cisplatin in advanced head and neck cancer (NCT03028766 and NCT02585973).

### Combining DNA Repair Targeting, Immunotherapy, and Radiotherapy

DNA repair proteins preserve the integrity of the genome; therefore, DNA repair targeting may enhance the tumor mutational burden, which may lead to the production of neoantigens and increased activity of anti-cancer T cells. Some clinical trials have been set to investigate the use of immune checkpoint inhibitors, notably by combining Dravulumab (anti-PD-L1) with PARP (NCT02484404), ATR (NCT02264678) or WEE1 inhibition (NCT02617277). To date, the interplay between radiation and the immune system is far from being completely deciphered, but several interesting facts have been reported. The cytotoxic action of radiotherapy on tumor cells provides T lymphocytes with tumor neoantigens and releases pro-inflammatory cytokines, thus promoting the immune response (137). The cell death inducing this type of immune response is called immunogenic cell death. Combining immunotherapy with radiotherapy (several recent trials: NCT02707588, NCT02952586, NCT02999087) could increase the ability to cause immunogenic cell death by removing locks that block the immune system (138). The non-overlapping toxicities of DNA repair targeting and immune checkpoint inhibitors render the use of combinations of these agents with radiotherapy appealing (14, 139).

## Conclusion

Targeted therapies are beginning to demonstrate activity across a number of tumor types. The most promising approach toward improving the efficiency of a treatment and gaining a reliable response is to develop therapy combinations that decrease the chance of resistance and to treat resistance when it emerges. There has been a considerable renewed emphasis on new targeted treatments such as radiosensitizers that do not cause overlapping dose-limiting toxicities. Selection of appropriate targeted agents represents a challenge. As predicted, during preclinical and clinical trials, particular attention was paid to proteins involved in DNA repair pathways. Various strategies have been explored, ranging from specific protein targeting to global inhibition, and many DNA repair inhibitors have been developed. Up until now, only a few of them have reached the clinical stage, while even fewer have been tested in combination with RT. The several clinical trials currently underway will tell whether these new compounds can be tolerable and efficient.

RT-induced lesions can be corrected by various DNA repair pathways. The intricacy of crosstalk between DNA repair pathways suggests that biomarker assays to determine the status of multiple DNA repair pathways could provide essential information on the sensitivity and resistance of cancer cells to repair inhibitors. Understanding these DNA repair pathways and identifying effective stratification biomarkers from the various DNA repair pathways that are specifically altered in some tumors would be required to characterize patients' responses to specific DNA repair inhibitors.

## Author Contributions

JB and EC analyzed the literature and wrote the manuscript. PV and MD gave important intellectual input and carefully revised the manuscript. All authors approved the final manuscript for submission.

### Conflict of Interest

MD is the cofounder of DNA Therapeutics/Onxeo, which is involved in DT01 development. The remaining authors declare that the research was conducted in the absence of any commercial or financial relationships that could be construed as a potential conflict of interest.
